# Long-term neurodevelopment outcomes of regional vs general anesthesia for infants undergoing inguinal herniorrhaphy: A protocol for systematic review and meta-analysis

**DOI:** 10.1097/MD.0000000000021669

**Published:** 2020-08-14

**Authors:** Tao Yuan, Wenming Yang, Lei Yang, Xueting Liu, Lie Yang, Yu Li

**Affiliations:** a Department of Anesthesiology; b Translational Neuroscience Center, Laboratory of Anesthesia and Critical Care Medicine; c Department of Gastrointestinal Surgery; d Department of Evidence-Based Medicine and Clinical Epidemiology, West China Hospital of Sichuan University, Chengdu, China.

**Keywords:** general anesthesia, inguinal herniorrhaphy, meta-analysis, neurodevelopment outcomes, protocol, regional anesthesia

## Abstract

**Background:**

Whether regional anesthesia (RA) offers better long-term neurodevelopment outcomes compared to general anesthesia (GA) to infants undergoing inguinal herniorrhaphy is still under heated debate. The aim of this meta-analysis is to compare the long-term neurodevelopment impact of RA with GA on infants undergoing inguinal herniorrhaphy.

**Methods:**

A systematic search of MEDLINE, EMBASE, PubMed, the Cochrane Central Register of Controlled Trials, clinicaltrials.gov and controlledtrials.com will be performed. Published eligible randomized controlled trials (RCTs) or quasi-RCTs (including abstracts) through May 20, 2020 with language limit of English will be enrolled in the meta-analysis. Two reviewers will independently conduct the procedures of study selection, data extraction, methodological quality assessment, and risk of bias assessment. The primary outcome is long-term neurodevelopmental state (at 2- and 5-year follow-up) as reflected in the Bayley and the Wechsler Preschool and Primary Scale of Intelligence (WPPSI) scales of infants development following surgeries. The secondary outcomes consist of satisfactory intraoperative infants immobility, duration of surgery, any anesthetic failure, the supplement of postoperative analgesia, postoperative apnea, and postoperative bradycardia. The pooled weighted mean differences (WMDs) or odds ratios (ORs) of each outcome measurement and relative 95% confident intervals (CIs) will be calculated. EndNote X8 (Clarivate Analytics) software will be applied to manage all citations. The Cochrane Review Manager version 5.3 software (RevMan 5.3) will be employed for statistical analysis.

**Discussion:**

This study will summarize scientific and practical evidence and provide evidence-based individualized decision-making guidance on anesthesia regimen for inguinal herniorrhaphy in infants.

**Registration:**

This protocol was registered with the International Platform of Registered Systematic Review and Meta-Analysis Protocols (INPLASY) on 17 June 2020 (registration number INPLASY202060064).

## 1 Introduction

Inguinal hernia (IH) is a common developmental defect in infants and children.^[1]^ IH requires early surgical repair to reduce the risk of incarceration, intestinal obstruction, and gonadal infarction.^[2]^ With improved anesthetic techniques and pediatric care management protocols, more infants with IH are presenting for surgery in early infancy.^[3]^

However, in addition to surgical complications, there is a concern that anesthetic agents may produce direct toxic effect on brain development of infants even after growing up. In 2016, the US Food and Drug Administration (FDA) warned that repeated or lengthy use of general anesthetic and sedation drugs during surgeries or procedures in children younger than 3 years may affect the development of children's brains.^[4]^ Experimental studies have demonstrated a correlation between the use of anesthetic agents and brain cell destruction.^[5]^ However, a nationwide cohort study in Denmark by Hansen et al suggested that differences in mental development indices and academic performance were no longer statistically significant between exposed (inguinal herniorrhaphy in infancy) and unexposed groups after adjusting for known confounders associated with poor neurosensory outcomes.^[6]^ More recently, the Pediatric Anesthesia Neurodevelopment Assessment (PANDA) study indicated that there were no statistically significant differences in intelligence quotient (IQ) scores in later childhood between healthy children with a single anesthesia exposure to general anesthesia (GA) before age 36 months and their healthy siblings with no anesthesia exposure.^[7]^ Despite increased anesthetic failure, relatively poorer intraoperative infant immobility, and presence of rare severe risk of spinal cord injury, regional anesthesia (RA) is seemingly considered a promising alternative to GA in infants undergoing inguinal herniorrhaphy by anesthesiologists and surgeons.

Whether RA offers better long-term neurodevelopment outcomes compared to GA to infants undergoing inguinal herniorrhaphy is still under heated debate. The results of this meta-analysis are expected to provide new evidences for more detailed individualized perioperative management for pediatric surgery.

## 2 Methods

This protocol was registered with the International Platform of Registered Systematic Review and Meta-Analysis Protocols (INPLASY) on 17 June 2020 (registration number *INPLASY202060064*; available from: https://inplasy.com/inplasy-2020-6-0064/). This protocol is established in compliance with the guideline of Preferred Reporting Items for Systematic Review and Meta-Analysis Protocols (PRISMA-P) checklist.^[8]^

### 2.1 Eligibility criteria

#### 2.1.1 PICO

P: infants undergoing inguinal herniorrhaphy;

I: regional anesthesia;

C: general anesthesia;

O: long-term neurodevelopment outcomes.

#### 2.1.2 Study design

Published eligible randomized controlled trials (RCTs) or quasi-RCTs (including abstracts) will be enrolled in the meta-analysis. The sequence generation, blinding, and allocation concealment should be explicitly described. Only articles originally written in English or translated into English will be considered.

#### 2.1.3 Types of participants

Infants who underwent inguinal herniorrhaphy with regional or general anesthesia within 60 weeks postpartum will be included.

#### 2.1.4 Comparison of interventions

Any form of regional anesthesia (including spinal, epidural, caudal, and local infiltration anesthesia, and regional nerve blockade) compared to general anesthesia (including various combinations of techniques of airway management and volatile or intravenous anesthetic agents for analgesia, sedation, and nerve muscle blockade) with or without regional analgesia which is left to the discretion of anesthesiologists.

#### 2.1.5 Outcome measures

The primary outcome is long-term neurodevelopmental state (at 2- and 5-year follow-up) as reflected in the Bayley and the Wechsler Preschool and Primary Scale of Intelligence (WPPSI) scales of infants development following surgeries. The secondary outcomes consist of satisfactory intraoperative infants immobility, duration of surgery, any anesthetic failure, the supplement of postoperative analgesia, postoperative apnoea, and postoperative bradycardia.

### 2.2 Information sources and search strategy

A systematic search of MEDLINE, EMBASE, PubMed, the Cochrane Central Register of Controlled Trials, clinicaltrials.gov and controlledtrials.com will be performed. The US “Society for Pediatric Research” and the European Society for Pediatric Research and Pediatric Anesthesia databases will be also searched. The relative references and network resources in the included literature will be further screened to find out the potential eligible ones. When multiple reports describing the same sample are published, the most recent or complete report will be included. All RCTs published in electronic databases before May 20, 2020 with language restricted in English will be included in this review study.

The Medical Subject Headings (MeSH), text words, and word variants for “infants,” “inguinal herniorrhaphy,” “inguinal hernioplasty,” “repair of inguinal hernia,” “inguinal hernia repair,” “regional anesthesia,” “spinal anesthesia,” “regional nerve blockade,” “general anesthesia,” “ananesthesia,” “neurodevelopment,” “neurological development,” and various combinations will be used in the searches. This search strategy will be modified to be suitable for other certain electronic databases.

### 2.3 Study selection and data collection

#### 2.3.1 Study selection

**Figure 1:**
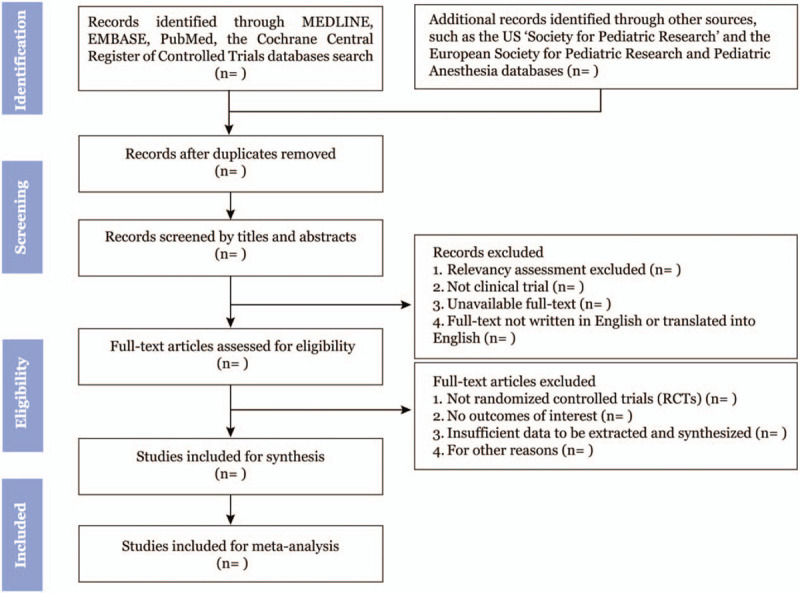
Flow diagram of study selection.

Two review authors (TY, WY) will screen all searched titles and abstracts independently for eligibility and relevance to this meta-analysis. EndNote X8 (Clarivate Analytics) software will be applied to manage all citations, as well as for duplicates removing. The study selection procedure is summarized in a PRISMA-P flow diagram as shown (Fig. 1). All studies meeting the exclusion criteria, such as case reports, letters, conference summaries, will be removed with recorded reasons firstly. Then full-texts of studies will be scrutinized with the inclusion criteria. Review authors will discuss any disagreements and involve a further party (YL) to help resolve remaining conflicts.

#### 2.3.2 Data extraction and collection

Two review authors (TY, LY) will extract relevant data from selected studies under cross check, including general characteristics (source, region, year of publication, title, first author, study type), data for methodological quality assessment, participants’ characteristics in each group (RA vs GA), median follow-up period, and all outcomes of interest. In addition, we will contact the corresponding author by e-mail to request for complete original data if necessary. Then we will enter the cross-checked data into the Cochrane Review Manager version 5.3 software (RevMan 5.3) for high-quality management and subsequent data synthesis.

### 2.4 Methodological quality assessment

The methodological quality will be evaluated by two review authors (WY, LY) according to RevMan 5.3 ‘Risk of Bias’ (RoB) assessment tool in terms of selection bias (method of randomization and allocation concealment), information bias (masking of outcome adjudicators), and bias in the analysis (intention to treat analysis and completeness of follow-up). Risk of bias for each study will be calculated using the criteria outlined in the Cochrane Handbook for Systematic Reviews of Interventions.^[9]^ The strength of the body of evidence will be graded into 3 levels, including “High Risk,” “Low Risk,” and “Unclear.” Disagreement between 2 independent reviewers will be solved by discussion and consulting the expert (XL) in Evidence-Based Medicine (EBM). The RoB table and graph will be drawn by RevMan 5.3.

### 2.5 Data synthesis and statistical analysis

The RevMan 5.3 software (The Nordic Cochrane Centre, The Cochrane Collaboration, Copenhagen, Denmark) will be employed for statistical analysis. Continuous data (scores of Bayley-III^[10]^ and WPPSI-III^[11]^ scale, duration of surgery) will be expressed as the weighted mean differences (WMDs) and relative 95% confidence intervals (CIs). Dichotomous data (satisfactory intraoperative infants immobility, any anesthetic failure, the supplement of postoperative analgesia, postoperative apnoea, postoperative bradycardia) will be expressed as the odds ratios (ORs) with 95% CIs. Statistical significance will be set at *P* < .05 to summarize the findings across the studies. Considering heterogeneity between studies, pooled analyses will be conducted with a random effect model (REM) rather than a fixed effect model (FEM). Statistical heterogeneity between studies will be evaluated using the chi-square (χ^2^) test and quantified with Cochrane's Inconsistency (*I*^2^)-statistic. We set 50% as a cut-off value, such that *I*^2^ > 50% are considered substantial heterogeneity. Sensitivity analysis and subgroup analysis will be set up to identify the sources of heterogeneity among the included studies.

### 2.6 Subgroup analysis and sensitivity analysis

Subgroup analyses will be performed to explore possible sources of heterogeneity. Subgroup analyses will be conducted based on sex, age, region, race, the use of preoperative sedatives, full-term pregnancy or premature and history of apnea in the preoperative period.

The sensitivity analysis will be performed to ensure the stability of measure effects of primary outcomes by removing one by one those studies with high risk of bias in terms of sample size, study design, heterogeneity qualities, and with non-informative prior distributions for the heterogeneity parameters. Non-robust results of primary outcomes identified by sensitivity analysis will be added to a descriptive analysis.

### 2.7 Publication bias

The possibility of publication bias will be estimated primarily by visual analysis of a funnel plot. If necessary, the Egger test will be applied to identify further potential publication bias.

### 2.8 Ethical approval and dissemination

The ethical approval is not required for the meta-analysis. The collected data will be stored for 3 years after completion of this study. The information obtained through data synthesis will be published in a scientific journal and publicly shared. Before being completely destroyed, data are available from the responsible authors on a reasonable request.

## 3 Discussion

An increasing number of infants receive surgical treatment and invasive procedures under anesthesia every year worldwide.^[12]^ Any potential neurocognitive risks of pediatric anesthesia are major scientific and public health issues. There is always a concern that exposure to GA in early infancy may produce harmful effect to developing human brain which shows developmental and behavioral disorders and learning disabilities.^[13]^

Currently, unlike adults, the optimal anesthetic regimen (RA or GA) for inguinal herniorrhaphy in infants is still uncertain. Some large human cohort studies have confirmed an association between GA at a younger age and subsequent neurodevelopmental deficits,^[14]^ but some of these studies are prone to bias. The General Anesthesia compared to Spinal anesthesia (GAS) study concluded that GA less than 1 hour (like most of inguinal herniorrhaphy surgeries) in early infancy would not alter neurodevelopmental outcome at age 5 years compared to awake-RA.^[15]^ Providing existing controversy on long-term neurodevelopment outcomes, we plan to conduct a meta-analysis to investigate the question. This study will summarize scientific and practical evidence and provide evidence-based individualized decision-making guidance on anesthesia regimen for infants undergoing inguinal herniorrhaphy.

## Author contributions

**Conceptualization:** Tao Yuan, Yu Li, Lie Yang.

**Software and data curation:** Wenming Yang, Lei Yang, Xueting Liu.

**Methodology and statistical analysis:** Tao Yuan, Yu Li, Lie Yang, Xueting Liu.

**Supervision and validation**: Yu Li, Lie Yang.

**Writing – original draft**: Tao Yuan, Wenming Yang.

**Writing – review & editing**: Yu Li, Lie Yang.
